# Emergence of multidrug-resistant *Candidozyma auris* in southern China: a multicenter study on genetic diversity and antifungal resistance

**DOI:** 10.3389/fmicb.2025.1689176

**Published:** 2025-10-10

**Authors:** Penghao Guo, Jingchun Fang, Ruizhi Wang, Weihong Lin, Yueting Jiang, Jun Long, Lingjuan Chen, Xudong Huang, Kang Liao, Yaqin Peng

**Affiliations:** ^1^Department of Clinical Laboratory, The First Affiliated Hospital of Sun Yat-sen University, Guangzhou, Guangdong, China; ^2^Department of Clinical Laboratory, Nansha Division of The First Affiliated Hospital, Sun Yat-sen University, Guangzhou, Guangdong, China; ^3^Department of Clinical Laboratory, The First Affiliated Hospital of Guangzhou Medical University, Guangzhou, Guangdong, China; ^4^Department of Laboratory Medicine, Microbiome Medicine Center, Zhujiang Hospital of Southern Medical University, Guangzhou, Guangdong, China; ^5^Department of Clinical Laboratory, Qingyuan People’s Hospital, The Sixth Affiliated Hospital of Guangzhou Medical University, Qingyuan, Guangdong, China; ^6^Department of Clinical Laboratory, Jieyang People’s Hospital, Jieyang, Guangdong, China

**Keywords:** *Candidozyma auris*, molecular epidemiology, fungal drug resistance surveillance, antifungal resistance, drug resistance mechanisms

## Abstract

**Purpose:**

*Candidozyma auris* (*C. auris*) is an emerging fungal pathogen that is resistant to multiple drugs and poses a serious threat to global health. This study aimed to investigate the genetic diversity and antifungal resistance profiles of *C. auris* in southern China.

**Methods:**

A total of 108 clinical *C. auris isolates* were collected from eight hospitals in Guangdong Province between January 2023 and January 2024. All the isolates were identified via matrix-assisted laser desorption ionization-time of flight mass spectrometry (MALDI-TOF MS). Phylogenetic analysis was conducted on the basis of the sequencing results of the *RPB1* and *D1/D2* genes. The mechanisms of resistance to fluconazole and echinocandins were investigated through sequencing of the *ERG11* and *FKS1* genes. Additionally, whole-genome sequencing was performed on echinocandin-resistant and echinocandin-sensitive isolates to analyse genetic homology among the strains.

**Results:**

All the strains were classified into two genetic clades, clade I and clade III, and all exhibited resistance to fluconazole. In the fluconazole-resistant strains, the amino acid substitutions Y132F and VF125AL were identified in the *ERG11* gene. The resistance rates to caspofungin, micafungin, and anidulafungin were 7.4, 7.4, and 3.7%, respectively. Among the eight echinocandin-resistant strains, amino acid substitutions (S639Y, W691L, and S639F) were found within the HS1 hotspot region of the *FKS1* gene. A phylogenetic tree was constructed on the basis of 403 SNPs and revealed two major clusters: Cluster A and Cluster B. Cluster A included the 16 isolates analyzed in this study. Cluster B consisted of 12 reference isolates retrieved from publicly available genomic databases.

**Conclusion:**

Genetic clade I and clade III *C. auris* strains are prevalent in southern China and present high levels of resistance to fluconazole. Controlling the spread of *C. auris* in this region presents significant challenges for public health management.

## 1 Introduction

*Candidozyma auris* (formally *Candida auris*), an emerging multidrug-resistant fungal pathogen, was first isolated in Japan in 2009 (Satoh et al., 2009). The incidence of *C. auris* infections has risen dramatically around the world (Du et al., 2020; Akinbobola et al., 2023; Chakrabarti and Sood, 2021). It can cause serious infections, with an associated mortality rate ranging from 30 to 60% (Azar et al., 2017; Lockhart et al., 2017). Importantly, *C. auris* is resistant to most currently available antifungal medicines, and some strains are resistant to all three types of antifungal medicines (Chaabane et al., 2019). In the United States, approximately 90% of *C. auris* isolates are resistant to fluconazole, approximately 30% are resistant to amphotericin B, and less than 2% are resistant to echinocandins (Centers for Disease Control and Prevention, n.d.).

Additionally, *C. auris* can persist in hospital environments and on surfaces for extended periods, along with its high transmission ability. This poses significant challenges for clinical diagnosis, prevention, and control (Chakrabarti and Singh, 2020; Chowdhary et al., 2017). Owing to its intrinsic resistance to multiple antifungal classes and its high potential for transmission, *C. auris* is often referred to as a “superbug.” Consequently, the World Health Organization has included *C. auris* in its list of fungal priority pathogens (WHO, 2022).

In 2018, Wang et al. reported the first case of a nondrug-resistant *C. auris* strain at Peking University People’s Hospital in Beijing, China (Wang et al., 2018). Tian et al. identified 15 cases of *C. auris* colonization or infection at a general hospital in Shenyang, China (Tian et al., 2018). Recently, our team reported seven cases of *C. auris* bloodstream infections in two hospitals in Guangdong Province, China (Peng et al., 2024). To date, multiple healthcare facilities in southern China have reported instances of *C. auris* colonization and infection. To understand the epidemiological characteristics of *C. auris* in southern China, this study analyzed clinical isolates from eight hospitals, with a focus on geographic distribution, phylogenetic relationships, and hotspot mutations related to antifungal drug resistance. This study includes the seven previously reported bloodstream isolates.

## 2 Methods

### 2.1 Clinical data and isolates

Between January 2023 and January 2024, 108 *C. auris* isolates were collected from eight hospitals in southern China. Duplicated isolates obtained from the same patient were excluded.

All the isolates were identified as *C. auris* by matrix-assisted laser desorption ionization time-of-flight mass spectrometry (MALDI-TOF MS) (bioMérieux, France) with the IVD knowledge base V3.2 according to the manufacturer’s instructions. Patient demographic and clinical data, such as sex, age, ward, underlying medical conditions, and antifungal treatments, were retrieved from the Hospital Information System.

### 2.2 Antifungal susceptibility testing

Antifungal susceptibility testing was performed using a commercial broth microdilution kit (Sensititre Yeastone™, Thermo Fisher Scientific). The antifungal agents tested included fluconazole (FLU), itraconazole (ITR), voriconazole (VOR), micafungin (MFG), caspofungin (CAS), anidulafungin (ANI), amphotericin B (AMB), 5-flucytosine (5-FC), and posaconazole (POS). *Candida krusei* (currently known as *Pichia kudriavzevii*) ATCC 6258 and *Candida parapsilosis* ATCC 22019 were used as quality control strains. The antifungal resistance of *C. auris* was interpreted according to the tentative minimum inhibitory concentration (MIC) breakpoints recommended by the US Centers for Disease Control and Prevention. Resistance was defined as an MIC ≥ 2 μg/mL for amphotericin B, ≥32 μg/mL for fluconazole, ≥2 μg/mL for caspofungin, ≥4 μg/mL for micafungin, and ≥2 μg/mL for anidulafungin.

### 2.3 Multilocus sequence typing

All strains were sequenced for the *D1/D2* and *RPB1* gene regions, and homology analysis was performed on the obtained sequences. The PCR and sequencing primers used are listed in Table 1. The procedure was carried out as follows: (1) The *D1/D2* and *RPB1* genes were amplified and sequenced. (2) Reference sequences of *D1/D2* and *RPB1* from geographically diverse strains were retrieved from the NCBI database. The gene accession numbers are provided in the supplementary material. (3) Sequences for both loci were concatenated for each strain. (4) Multiple sequence alignment and phylogenetic analysis were conducted using MEGA 7.0. A maximum likelihood phylogenetic tree was constructed with 1,000 bootstrap replicates.

**Table 1:** List of primers used for PCR amplification and sequencing of target genes.

Primer name	Sequence (5′-3′)	Target gene/purpose
*ERG11aF*	ATGGCCTTGAAGGACTGCATCGT	*ERG11* PCR and sequencing
*ERG11aR*	TTAGTAAACACAAGTCTCTCTTTTCTCCCA	
*CRS_HS1F*	GCCATCTCGAAGTCTGCTCA	*FKS1* PCR and sequencing
*CRS_HS1R*	TGACAATGGCATTCCACACCT	
*CRS_HS2F*	GCGAGAACCTTGGCTCAAA	
*CRS_HS2R*	ATGGCAAGAAGTCAGCCATGA	
*CRS-RPB1F*	CACCTCTCGCAGTATCGTTG	*RPB1* PCR and sequencing
*CRS-RPB1R*	CCCGTCTTTCACATTGGTTT	
*NL1*	GCATATCAATAAGCGGAGGAAAAG	*D1/D2* PCR and sequencing
*NL4*	GGTCCGTGTTTCAAGACGG	

### 2.4 *ERG11*/*FKS1* gene sequencing and analysis

The *ERG11* gene was sequenced in fluconazole-resistant isolates. Additionally, the hotspot regions HS1 and HS2 of the *FKS1* gene were analyzed in isolates showing reduced echinocandin susceptibility. Details of the primers used are provided in Table 1.

Sequence analysis involved assembling and aligning the obtained sequences with reference sequences from the NCBI database: *FKS1* (accession numbers: OQ378936.1 and OQ378944.1) and *ERG11* (accession numbers: MK294627 and MK294628). Reference sequences were derived from both fluconazole-susceptible and fluconazole-resistant strains, as well as from echinocandin-susceptible and echinocandin-resistant isolates. Potential nucleotide variations were identified using MEGA 7.0 software.

### 2.5 Whole-genome sequencing and phylogenetic analysis

Whole-genome sequencing was performed on 16 isolates using the TELL-seq library construction and sequencing methods. Among these strains, eight isolates exhibited echinocandin resistance, whereas the remaining eight were echinocandin susceptible. The 8 echinocandin-susceptible isolates were selected from hospitals where echinocandin-resistant strains had been isolated. During isolate selection, susceptible strains were randomly chosen according to the geographic distribution of the echinocandin-resistant strains.

TELL-seq libraries were constructed using the TELL-Seq Microbial Library Prep Kit (Universal Sequencing Technology), with 0.1 ng of genomic DNA per isolate. Library quantification was carried out using the Qubit 1X dsDNA HS Assay Kit (Thermo Fisher Scientific), followed by pooling and sequencing on an Illumina NovaSeq 6000 instrument, generating 2 × 150 paired-end reads along with 18-cycle Index 1 and 8-cycle Index 2 reads, according to the manufacturer’s protocols.

Single-nucleotide polymorphisms (SNPs) were identified using the Haplotype Caller module of the Genome Analysis Toolkit (GATK). The resulting gVCF files were merged via CombineGVCFs, and the SNPs were filtered on the basis of a variant quality score (<30) and quality by depth (<10). SNP calling was further refined using the Snippy v4.6.0 pipeline.

This study used the *C. auris* reference genome (GenBank accession number: GCA_014217455.1) for sequence alignment. The whole-genome sequencing data of twelve *C. auris* isolates (clade III) from public databases were included in the analysis. A maximum likelihood phylogenetic tree was constructed using SNP data with IQ-TREE software, with the TVMe + ASC + R3 model applied. Ultrafast bootstrap analysis was performed with 1,000 replicates.

## 3 Results

### 3.1 Clinical characteristics

The study included 108 patients with a median age of 64.3 years (range: 22–83 years). A total of 54.6% of the patients were admitted from the intensive care unit (ICU), and the male-to-female ratio was 2.2:1. The mean length of hospital stay was 53 days (range: 7–267 days). The median time from admission to the first identification of *C. auris* was 29.5 days (range: 4–129 days).

Prior to positive cultures for *C. auris*, 73.1% of the patients had undergone mechanical ventilation, 89.7% had been administered broad-spectrum antibiotics, and 91.0% had indwelling urinary catheters. Notably, 30.8% of patients were colonized or infected at multiple anatomical sites. At discharge, 34.7% of the patients had cleared the organism. Sputum and bronchoalveolar lavage fluid (BALF) are the most common sources of clinical samples. The detailed demographic and clinical characteristics are summarized in Table 2.

**Table 2:** Demographic and clinical characteristics of the 108 patients.

Variables	Number (cases)	Percentage (%)
Female	34	31.5
Male	74	68.5
Department distribution		
ICU	59	54.6
Medical ward	32	29.6
Surgical ward	15	13.9
Emergency ward	2	1.9
Distribution of specimen types		
Sputum/BALF	36	34.6
Urine	34	32.7
Blood	10	9.6
Drainage fluid	9	8.7
Catheter	8	7.7
Wound secretion	7	6.7
Prognosis		
Died	19	17.6
Discontinued treatment	12	11.1
Improved	77	71.3

ICU, intensive care unit; BALF, bronchoalveolar lavage fluid.

### 3.2 Antifungal susceptibility testing

**Figure 1:**
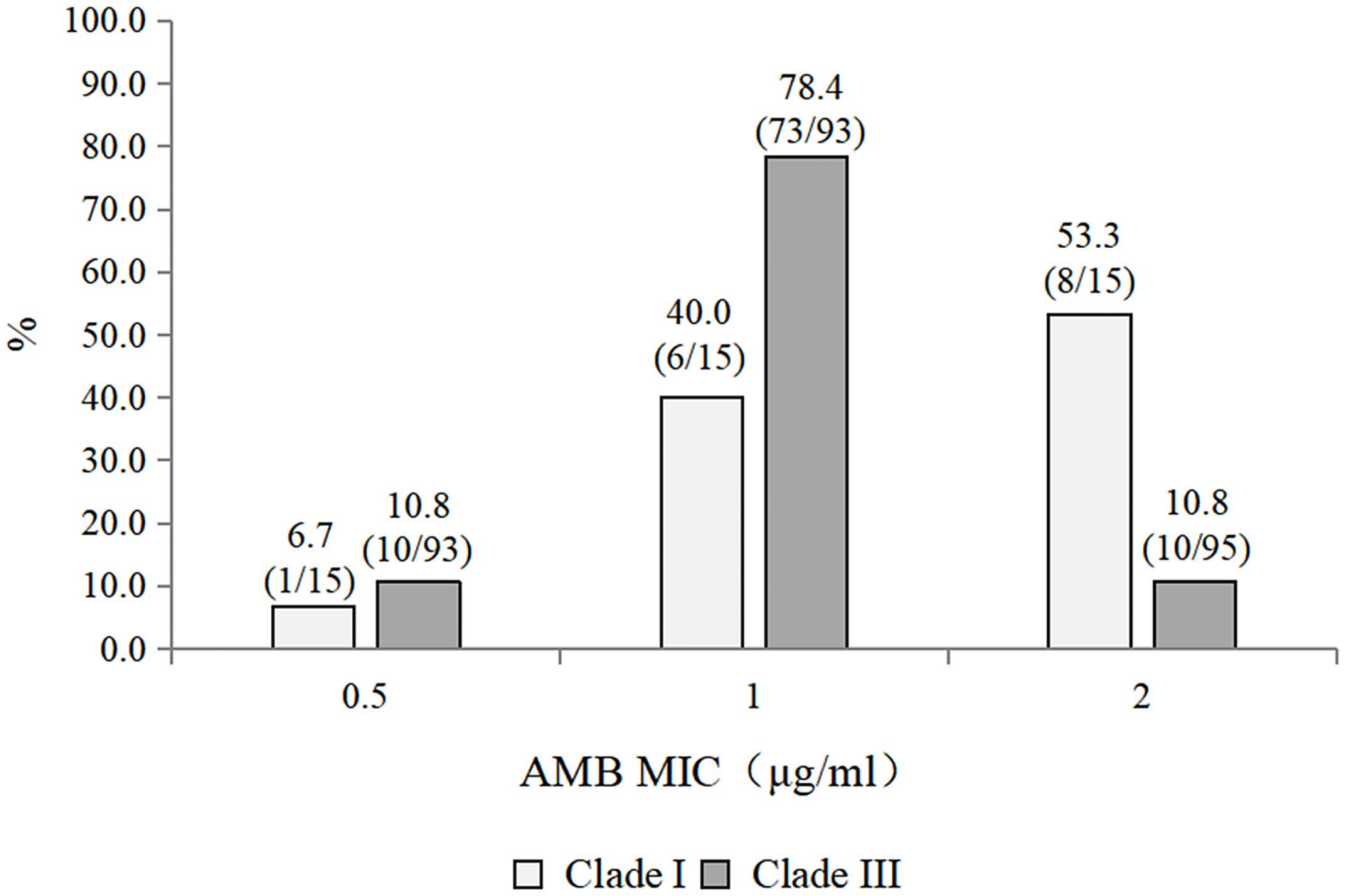
Distribution of amphotericin B MICs among clades I and III *Candidozyma auris* strains.

The antifungal susceptibility profiles of all the isolates are summarized in Table 3. All the isolates were resistant to fluconazole. The resistance rates to caspofungin, micafungin, and anidulafungin were 7.4, 7.4, and 3.7%, respectively. The resistance rate to amphotericin B was 16.7%. Among the triazoles, posaconazole presented the lowest geometric mean (GM) values. Caspofungin, micafungin, and anidulafungin displayed comparable MIC_50_ values, GM values, and MIC distributions. Compared with the Clade III strains (10/93, 10.8% resistant), the 15 Clade I strains exhibited a higher rate of amphotericin B resistance (8/15, 53.3% resistant) (Figure 1). Notably, one strain exhibited multidrug resistance and was concurrently resistant to amphotericin B, echinocandins, and fluconazole.

**Table 3:** *In vitro* antifungal susceptibility profile of all the isolates.

Drug	MIC (μg/ml)										Resistance rate	MIC_50_ (μg/ml)	Range (μg/ml)	GM (μg/ml)
	0.03	0.06	0.125	0.25	0.5	1	2	4	≥8	≥32				
ANI	0	1	79	14	6	0	4	3	1	0	3.7%	0.125	0.06–8	0.15
CAS	1	15	63	16	5	0	4	2	2	0	7.4%	0.125	0.03–8	0.16
MIF	1	12	77	5	5	0	0	1	7	0	7.4%	0.125	0.06–8	0.14
FLU	0	0	0	0	0	0	0	0	0	108	100%	128	32–256	93.21
AMB	0	0	0	0	11	79	18	0	0	0	16.7%	1	0.5–2	1.05
5-FC	0	50	50	8	0	0	0	0	0	0	/	0.12	0.06–0.25	0.09
VOR^c^	0	0	0	1	56	41	9	0	1	0	/	0.5	0.5–2	0.64
POS^c^	17	61	27	1	0	0	0	0	2	0	/	0.06	0.015–0.25	0.07
ITR^c^	0	5	63	37	1	0	0	0	2	0	/	0.125	0.06–0.5	0.15

MIC, minimum inhibitory concentration; MIC50, MIC at which 50% of test isolates were inhibited; GM, geometric mean MICs; ANI, anidulafungin; CAS, caspofungin; MIF, micafungin; FLU, fluconazole; AMB, amphotericin B; 5-FC, 5-flucytosine; VOR, voriconazole; POS, posaconazole; ITR, itraconazole.

### 3.3 Multilocus sequence typing

**Figure 2:**
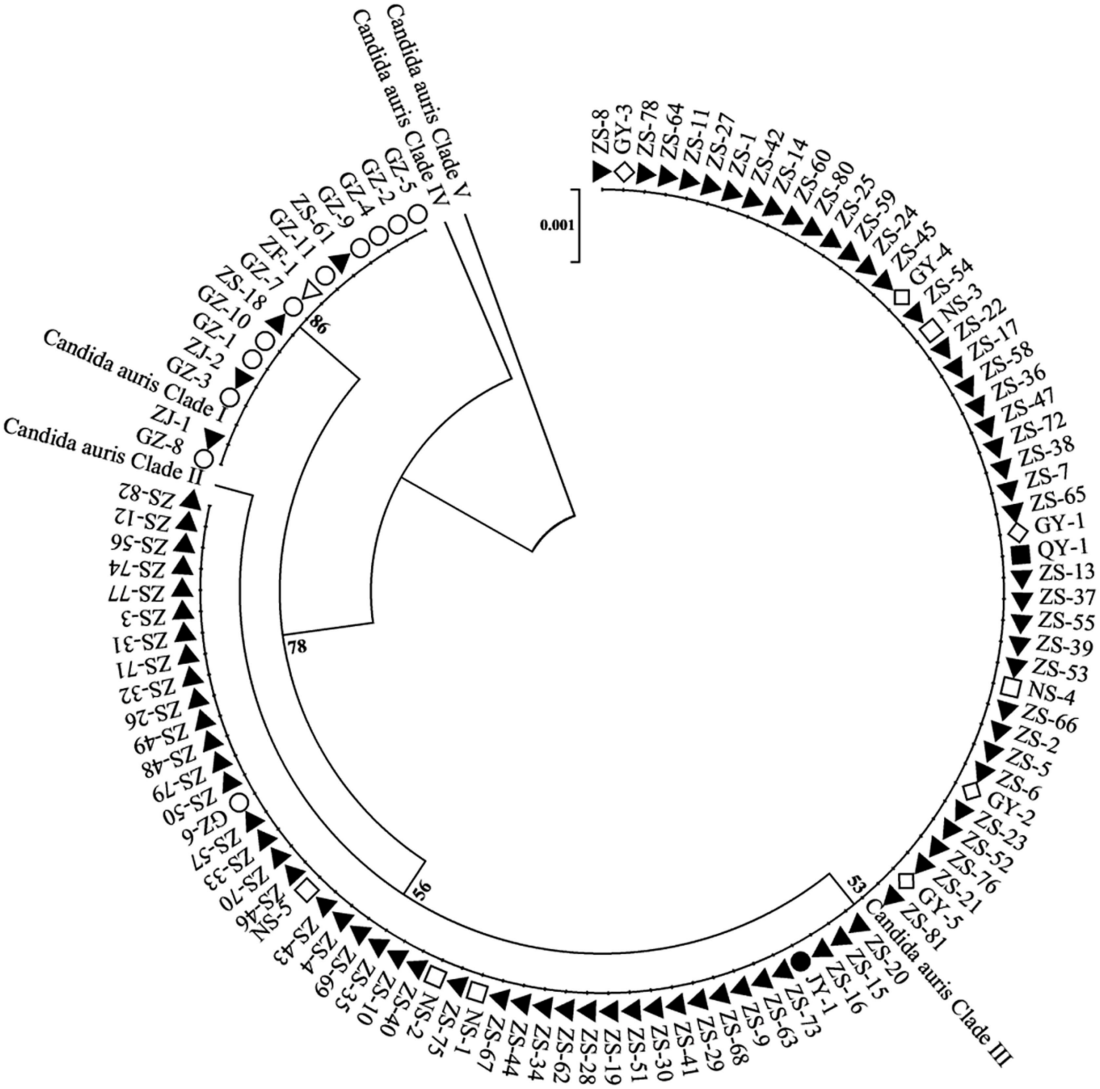
Phylogenetic relationships among *C. auris* isolates from multiple healthcare facilities. This phylogenetic tree illustrates the genetic relatedness of *C. auris* isolates based on the RPB1 and D1/D2 sequences. Each node represents an individual isolate. Different shapes and colors denote different healthcare facilities. The dendrogram shows that the strains are divided into two geographical clades: clade I (South Asian clade) and clade III (South African clade) and with the majority of the strains belonging to the clade III (South African clade).

On the basis of the sequencing analysis of the *RPB1* and *D1/D2* regions, 108 strains were grouped into two distinct genetic clades (clades I and III). Specifically, 93 strains (86.1%) belonged to clade III, whereas 15 strains (13.9%) were classified into clade I. Notably, the coexistence of clade I and clade III strains was observed in two hospitals. The phylogenetic relationships among the 108 *C. auris* strains were inferred using sequences from the *RPB1* and *D1/D2* regions and are presented in Figure 2.

### 3.4 *ERG11* and *FKS1* mutation analysis

**Figure 3:**
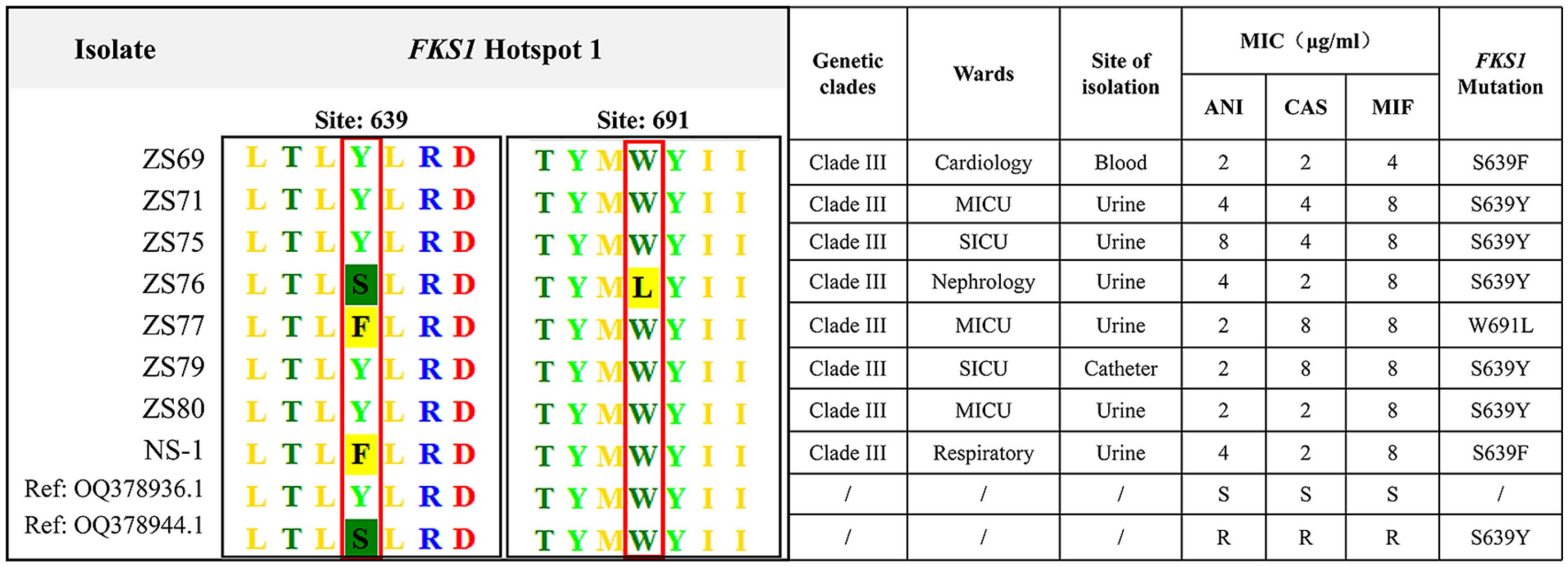
Clinical characteristics and *FKS1* mutations in eight echinocandin-resistant *C. auris* strains. The eight echinocandin-resistant isolates were obtained from two hospitals. All eight isolates belonged to clade III, of which six were isolated from urine specimens. The detected mutation sites, including S639Y, W691L, and S639F, were all located in the FKS1 gene.

Given that all 108 isolates exhibited fluconazole resistance, we performed ERG11 gene sequencing on all strains. All strains harbored mutations in the *ERG11* gene. The VF125AL amino acid substitution was present in all the clade III strains, whereas the Y132F substitution was exclusively detected in the clade I strains. Notably, no isolate exhibited both *ERG11* mutations at the same time. Additionally, eight echinocandin-resistant isolates carried mutations in the *FKS1* gene. Specific amino acid substitutions (S639Y, W691L, and S639F) were detected in the HS1 hotspot region of the *FKS1* gene. No mutations were detected in the HS2 hotspot region. Among these, five isolates exhibited the S639Y mutation, two carried the S639F mutation, and one contained the W691L mutation. All eight echinocandin-resistant strains belonged to clade III, with seven originating from the same hospital. Six isolates were recovered from urine cultures, one from a blood culture, and one from a catheter tip culture. The clinical characteristics and *FKS1* gene mutations of the eight echinocandin-resistant strains are summarized in Figure 3.

### 3.5 Phylogenetic analysis of 16 echinocandin-resistant and -susceptible strains

**Figure 4:**
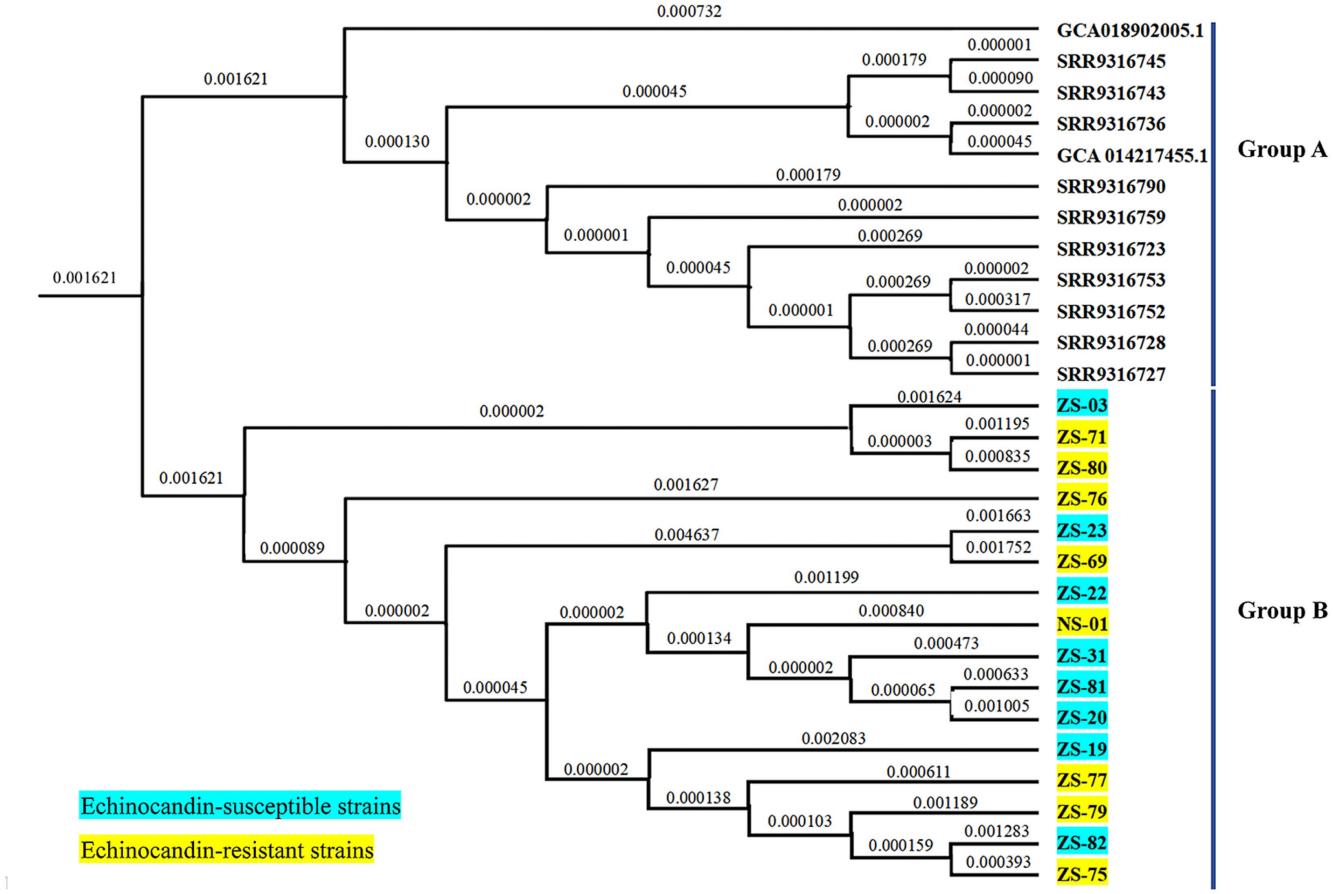
Phylogenetic tree of *C. auris* isolates from this study and selected isolates reported in China. Cluster A comprised 16 isolates collected from patients in Southern China between 2021 and 2023. Cluster B included 12 reference isolates obtained from publicly available genomic databases.

The draft genome assemblies of the 16 isolates ranged in size from 12.3 to 14.7 Mb, with an average of 23 ± 4 scaffolds. The GC content was 45.10% ± 0.03%, the scaffold N50 value was 1824.2 ± 369.1 kilobases (kb), and the coverage depth was 722 × ± 190 × on the basis of quality-trimmed reads. A phylogenetic tree was constructed on the basis of 403 SNPs and revealed two major clusters: Cluster A and Cluster B (Figure 4). Cluster A included the 16 echinocandin-resistant and -susceptible isolates analyzed in this study, all of which were collected from patients in Southern China between 2021 and 2023. Cluster B consisted of 12 reference isolates retrieved from publicly available genomic databases. These external isolates serve as a phylogenetic context, highlighting the genetic distinctness of the locally circulating strains in Cluster A.

## 4 Discussion

The clinical features of *C. auris* infections identified in this study are consistent with those reported in other studies (Du et al., 2022). First, invasive infections are relatively uncommon, with the majority of isolates obtained from nonsterile sites such as urine, sputum, or bronchoalveolar lavage fluid. Second, the majority of affected patients are elderly individuals who have been admitted to ICUs. These patients often undergo various medical interventions, including urinary catheterization, the administration of broad-spectrum antibiotics, and mechanical ventilation.

This study identified two genetic clades of *C. auris* (clade I and clade III) on the basis of sequencing analysis of *RPB1* and *D1/D2*. This distribution is consistent with epidemiological reports from other regions in China (Bing et al., 2024). The first reported case of *C. auris* infection in Guangdong Province was associated with travel, as the patient had received medical care at a hospital in South Africa, where Clade III strains are predominant (Szekely et al., 2019). Furthermore, interhospital patient transfers have contributed to dissemination in multiple healthcare facilities. In southern China, Clade I and Clade III of *C. auris* are co-circulating, which is different from other regions in the country. Notably, we found both Clade I and Clade III strains present simultaneously in the same hospitals—this finding has not been reported in any published multicenter or single-center studies from mainland China. The coexistence of these two clades suggests a more complex and diverse epidemiological landscape for *C. auris* in southern China.

In our study, all isolates were found to be resistant to fluconazole. The resistance rates for caspofungin, micafungin, and anidulafungin were 7.4, 7.4, and 3.7%, respectively. The resistance rate to amphotericin B was 16.7%. One strain exhibited multidrug resistance, showing resistance to amphotericin B, echinocandins, and fluconazole; it was isolated from a urine sample and caused a urinary tract infection in the patient. According to MLST (multilocus sequence typing) results, this strain belongs to clade III and carries mutations in both the *ERG11* and *FKS1* genes. Ultimately, the patient discontinued treatment.

Consistent with the well-documented high level of fluconazole resistance (Du et al., 2022), all strains exhibited fluconazole resistance. Among the azole antifungal agents tested, posaconazole presented the lowest MIC, followed by itraconazole. In contrast, the voriconazole MICs were significantly greater than those observed for both posaconazole and itraconazole. This finding is consistent with previously published data (Gaitán et al., 2017; Larkin et al., 2017; Calvo et al., 2016; Morales-López et al., 2017; Chowdhary et al., 2018). For example, a study from Colombia reported that 23.5% of isolates were nonsusceptible to voriconazole (MIC ≥ 2 μg/mL), whereas all isolates identified in Spain and Venezuela presented voriconazole MICs of ≥2 μg/mL (Gaitán et al., 2017; Calvo et al., 2016; Morales-López et al., 2017). In our current study, 8.7% of the isolates were found to be nonsusceptible to voriconazole.

It should be noted that there are currently no *C. auris*-specific antifungal susceptibility breakpoints established. The breakpoints currently used are those defined by U. S. Centers for Disease Control and Prevention (CDC), based on breakpoints for closely related *Candida* species and expert opinion. At present, the correlation between microbiological breakpoints and clinical treatment outcomes remains unknown. Besides, for other azoles such as posaconazole, itraconazole, and voriconazole, no validated clinical breakpoints are currently available for *C. auris*. Consider using fluconazole susceptibility as a surrogate for second generation triazole susceptibility assessment. However, isolates that are resistant to fluconazole may respond to other triazoles occasionally. The decision to treat with another triazole will need to be made on case-by-case basis.

Fluconazole and other triazole antifungal agents exert their antifungal activity by competitively inhibiting sterol 14α-demethylase, a key enzyme in the ergosterol biosynthesis pathway. A common mechanism of fluconazole resistance involves mutations in the *ERG11* gene, which encodes this essential enzyme. The VF125AL, Y132F, and K143R mutations have been frequently observed in fluconazole-resistant *C. auris* isolates (Rybak et al., 2022). Moreover, these mutations are associated with specific genetic clades (Lockhart et al., 2017). The VF125AL substitution was exclusively detected in clade III strains, whereas the Y132F substitution was exclusively detected in clade I and IV strains (Chow et al., 2020; Kwon et al., 2019). In our study, a consistent pattern was observed: all the clade III strains carried the VF125AL substitution, whereas all the clade I isolates harbored the Y132F mutation.

In contrast to the high amphotericin B resistance rate of 43% reported among *C. auris* strains in the United States, most isolates in China have exhibited low MICs for amphotericin B (Du et al., 2022). For example, Tian et al. reported a resistance rate of only 1.1% (1 out of 93) in Shenyang, China (Tian et al., 2021). However, the amphotericin B resistance observed in our study was greater. This discrepancy may be due to differences in the geographic clade distributions of the studied isolates. Specifically, all strains in Tian et al.’s study belonged to clade III, whereas our study included clade I and clade III strains. Emerging evidence suggests significant geographic variation in amphotericin B resistance rates among *C. auris* strains (Sanyaolu et al., 2022). Studies have shown that clade I has a 50% resistance rate to AMB—the highest rate in its clades. In contrast to the high amphotericin B resistance rate, clade III remains highly susceptible to AMB (da Silva et al., 2025). Previous studies suggest that putative loss-of-function mutations in *ERG6*, *ERG3,* and *ERG11* can cause acquired AMB resistance in *C. auris* (Rybak et al., 2022; Carolus et al., 2021). However, as many AMB resistance isolates of *C. auris* exhibit lower levels of resistance (MIC 2–4 mg/L), and the vast majority are observed to lack mutations in ergosterol-biosynthesis genes, it appears likely that other mechanisms of AMB resistance remain to be identified (Sharma and Kadosh, 2023).

Fortunately, resistance to echinocandins remains relatively rare among *C. auris* isolates, suggesting that these drugs are suitable as first-line therapeutic agents. However, clinicians should be aware that echinocandin-resistant *C. auris* isolates have been reported. Of particular concern is the emergence of echinocandin-resistant strains in Guangdong Province within approximately 1 year of the initial detection of *C. auris*. The rapid emergence of echinocandin resistance in this region, occurring within approximately 1 year of the initial detection of *C. auris*, highlights the notable ability of this pathogen to acquire resistance. This phenomenon has been reported in studies, where strains that were initially susceptible developed echinocandin resistance (Rhodes et al., 2018; Centers for Disease Control and Prevention, 2017; Spruijtenburg et al., 2023). Bram Spruijtenburg et al. described a case in which resistance to micafungin developed after only 4 days of treatment, despite initial susceptibility (Spruijtenburg et al., 2023).

In this study, among the 8 patients with echinocandin-resistant isolates, the initial isolates from 2 patients were susceptible to echinocandins. Previous investigations into *C. auris* and other clinically relevant *Candida* species have demonstrated higher rates of echinocandin resistance among isolates recovered from urine than among those from other anatomical sources (Adams et al., 2018; Sharma et al., 2022). Consistent with these findings, the majority of the echinocandin-resistant isolates in our study were obtained from urine samples. This trend may be attributed to the low urinary concentrations of echinocandin drugs. For example, urinary excretion accounts for only 1.4% of the plasma concentration of caspofungin, whereas the rates of excretion from micafungin and anidulafungin are 0.7% and less than 0.1%, respectively (Denning, 2003). These subtherapeutic concentrations in urine are insufficient to effectively clear *C. auris* infection or colonization but may promote the selection and emergence of resistant strains (Spruijtenburg et al., 2023).

The primary mechanism of echinocandin resistance in *Candida* species involves the acquisition of mutations in the *FKS* genes, which encode the catalytic subunits of 1,3-β-D-glucan synthase, a key enzyme targeted by these antifungal agents. These mutations can lead to structural changes in the 1,3-β-D-glucan synthase complex, reducing its susceptibility to echinocandins. To date, three major amino acid substitutions at position S639 within the *FKS1* gene have been identified in echinocandin-resistant *C. auris* isolates (Rybak et al., 2022; Denning, 2003; Kordalewska et al., 2018; Berkow and Lockhart, 2018; Rhodes et al., 2018). In line with these findings, our study confirms that the S639 (serine) amino acid position serves as the primary molecular determinant of echinocandin resistance in these strains.

Furthermore, whole-genome homology analysis revealed that the echinocandin- resistant strains in this study are phylogenetically more closely related to local susceptible isolates than to those from other geographic regions. The close phylogenetic clustering of echinocandin-resistant isolates within the same healthcare facility indicates a potential for nosocomial transmission or local development of resistance. However, the limited number of sequenced isolates and the absence of broader regional genomic data highlight the need for ongoing genomic surveillance.

This study represents the first comprehensive characterization of the epidemiological features of *C. auris* in southern China. However, the absence of whole-genome sequencing (WGS) data for all the isolates limits our ability to perform a detailed phylogenetic analysis of strain evolution. Consequently, the current findings lack sufficient molecular epidemiological evidence to delineate potential transmission routes between healthcare facilities. In addition, this study did not conduct in-depth analysis or discussion on mortality rates, clearance rates, and treatment regimens. Future studies should aim to elucidate the transmission dynamics of *C. auris*, investigate the mechanisms underlying resistance evolution, and assess their implications for clinical management and infection control practices.

## Data Availability

All the data generated or analyzed during this study are included in this article.
